# Relationship between age and handgrip strength: Proposal of reference values from infancy to senescence

**DOI:** 10.3389/fpubh.2022.1072684

**Published:** 2023-01-26

**Authors:** Rossana Gómez-Campos, Rubén Vidal Espinoza, Miguel de Arruda, Enio Ricardo Vaz Ronque, Camilo Urra-Albornoz, Juan Carlos Minango, Fernando Alvear-Vasquez, Christian de la Torre Choque, Luis Felipe Castelli Correia de Campos, Jose Sulla Torres, Marco Cossio-Bolaños

**Affiliations:** ^1^Departamento de Diversidad e Inclusividad Educativa, Universidad Católica del Maule, Talca, Chile; ^2^Faculty of Education, Psychology and Sport Sciences, University of Huelva, Huelva, Spain; ^3^Universidad Católica Silva Henriquez, Santiago, Chile; ^4^Universidad Estadual de Campinas, São Paulo, Brazil; ^5^Departamento de Educação Física (DEF), Universidade Estadual de Londrina, Londrina, Brazil; ^6^Escuela de Ciencias del Deporte, Facultad de Salud, Universidad Santo Tomás, Santiago, Chile; ^7^Instituto Tecnológico Universitario Ruminahui, Sangolqui, Ecuador; ^8^Facultad de Educación, Universidad Autónoma de Chile, Talca, Chile; ^9^Universidad San Ignacio de Loyola, Lima, Perú; ^10^Departamento de Ciencias de la Educación, Facultad de Educación y Humanidades, Universidad del Bio-Bio, Chillán, Chile; ^11^Universidad Católica de Santa María, Arequipa, Perú; ^12^Departamento de Ciencias de la Actividad Física, Universidad Católica del Maule, Talca, Chile

**Keywords:** dynamometer, hand grip strength, percentiles, infancy, senescence

## Abstract

**Introduction:**

Measurement of hand grip strength (HGS) has been proposed as a key component of frailty and has also been suggested as a central biomarker of healthy aging and a powerful predictor of future morbidity and mortality.

**Objectives:**

(a) To determine whether a nonlinear relationship model could improve the prediction of handgrip strength (HGS) compared to the linear model and (b) to propose percentiles to evaluate HGS according to age and sex for a regional population of Chile from infancy to senescence.

**Methods:**

A cross-sectional descriptive study was developed in a representative sample of the Maule region (Chile). The volunteers amounted to 5,376 participants (2,840 men and 2,536 women), with an age range from 6 to 80 years old. Weight, height, HGS (right and left hand) according to age and sex were evaluated. Percentiles were calculated using the LMS method [(L (Lambda; asymmetry), M (Mu; median), and S (Sigma; coefficient of variation)].

**Results and discussion:**

There were no differences in HGS from 6 to 11 years of age in both sexes; however, from 12 years of age onwards, males presented higher HGS values in both hands (*p* < 0.05). The linear regression between age with HGS showed values of *R*^2^ = 0.07 in males and *R*^2^ = 0.02 in females. While in the non-linear model (cubic), the values were: *R*^2^ = 0.50 to 0.51 in men and *R*^2^ = 0.26 in women. The percentiles constructed by age and sex were: P5, P15, P50, P85, and P95 by age range and sex. This study demonstrated that there is a nonlinear relationship between chronological age with HGS from infancy to senescence. Furthermore, the proposed percentiles can serve as a guide to assess and monitor upper extremity muscle strength levels at all stages of life.

## Introduction

Hand grip strength (HGS) is the amount of static force that the hand can generate around the dynamometer. It is defined as the ability of the hand to grasp objects between the thumb and fingers (1). It is characterized by completing a maximal isometric grip strength task, in which individuals squeeze a grip dynamometer with maximal effort for a short period of time and then the contracted musculature is relaxed (2).

Overall muscle strength is conveniently assessed by measuring (HGS) with a hand grip dynamometer (3). This equipment objectively measures upper extremity isometric strength (4) and is considered a prime candidate for use in routine medical examinations given its simplicity and low cost for assessing isometric strength in children, youth and adults (5).

Measurement of HGS has been proposed as a key component of frailty phenotypes and has also been suggested as a central biomarker of healthy aging and a powerful predictor of future morbidity and mortality in both young, as well as older adult populations (6, 7).

Indeed, measures of muscle strength and physical performance are increasingly used for research and practice. It is timely to review and identify appropriate tools for their assessment (8), so norm-referenced percentiles are an alternative to help interpret the performance of an individual compared to a reference population (9).

In that sense, reference ranges for HGS from infancy to senescence have been reported in several studies in high-income countries such as England (10) Canada (11) and the United States (12, 13). These scales are comparable and measurable to aid in interpetation of test results and decision making (13), such as the reference values proposed by the Center for Disease Control and Prevention [CDC] (14), developed from the United States National Health and Nutrition Examination Survey (NHANES).

These references are very scarce in South America, such as the study developed in Colombia (15). However, in Chile, as far as is known, no studies have been identified that cover a wide age range that would allow the assessment of HGS from 6 to 80 years of age, as has been observed in previous studies.

In general, reference values should be interpreted using specific ranges according to geographic region, ethnicity (16), age, sex, height (7). This is due to the fact that, not all country populations present similar characteristics (social, economic, cultural, demographic, nutritional and anthropometric). Therefore, their trajectories usually present varied levels of muscular strength performances, being higher in men in relation to women. These differences appear during the stage of growth and maturation, passing through youth, middle adulthood and old age) (10, 11).

Consequently, studying HGS from infancy to senescence is relevant, due to its ability to predict skeletal muscle strength throughout life, as studies generally use linear regression models to predict HGS (1, 17, 18). However, it is possible that there is a nonlinear relationship between age and HGS, as the cubic model could improve the predictive power of isometric strength when it is intended to be analyzed from infancy to senescence.

Therefore, the objectives of the study were (a) to determine whether a nonlinear relationship model could improve the prediction of HGS compared to the linear model and (b) to propose percentiles to assess HGS according to age and sex for a regional population of Chile from infancy to senescence.

## Methodologic

### Type of study and sample

A descriptive cross-sectional study was developed in a representative sample of the Maule region (Chile). The details of the sampling process were described in a previous study (19). Sampling was probabilistic (random). The volunteers amounted to 5,376 participants (2,840 males and 2,536 females), with an age range of 6 to 80 years old. They were recruited from public schools (schoolchildren aged 6 to 17 years), public and private universities (aged 18 to 30 years) and middle-aged and older adults from social programs offered by the Municipality of Talca (Maule Region). Maule is located in the seventh region of Chile 230 km south of the capital Santiago and the Development Index (HDI) for 2018 was 0.872, while for the country it was 0.847 (20).

Regarding the socio-demographic indicators of the Maule region (Chile), according to the Ministerio de Desarrollo Social Chile (21), there are small differences in relation to the capital of chile (Santiago). For example, the average years of schooling in the Maule region are around ~9.8 years, the employment rate is 55.7%, unemployment rate 6.9%, state social welfare 55.7%, private social welfare 5.9%. While, in the capital Santiago, the average years of schooling are ~11.6 years, the employment rate is 58.9%, unemployment rate 6.9%, state social security 71.0%, and private social security 21.3%.

All study participants signed the informed consent form, in which they authorized the anthropometric measurements and the assessment of HGS. For those under 18 years of age, it was the parents and/or guardians who signed the informed consent. Participants who were of a nationality other than Chilean were excluded, as well as those who presented some type of physical disability (that prevented them from being able to look after themselves). People who previously reported stroke, spinal cord or brain injury, or upper extremity disabilities were also excluded from the study.

The research was conducted from 2015 to 2018 and was developed according to the Declaration of Helsinki for human beings and had the approval of the University Ethics Committee (UA-238-2014).

### Techniques and procedures

A team of 6 evaluators was formed to collect data from the study sample. The anthropometric variables and the HGS were collected in schools, universities, and in the facilities of the social programs of the Municipality of Talca. This procedure was carried out from Monday to Friday from 8:30 am to 12:30 pm. Data such as age and date of birth were collected from the registration forms of each institution.

Anthropometric measurements of weight and height were evaluated according to the recommendations of Ross and Marfell-Jones (22). All participants wore as little clothing as possible (shorts, T-shirt, and bare feet). Body weight (kg) was measured using an electric scale (Tanita, Glasgow, UK, Ltd), accurate to 100g and with a scale from 0 to 150 kg. Height was measured in the standing position and according to the Frankfort plane (23). A portable stadiometer (Seca Gmbh and Co. KG. Hamburg, Germany), with an accuracy of 0.1 mm, and a scale of 0 to 205 cm, was used. Both anthropometric measurements were evaluated 2 times, where the relative technical error of measurement (TEM%) was 1.2%.

The HGS of both hands (right and left) was assessed by dynamometry. A JAMAR hydraulic dynamometer (brand name) (Hydraulic Hand Dynamometer^®^ Model PC-5030 J1, Fred Sammons, Inc., Burr Ridge, IL: USA) was used. This equipment has an accuracy of 0.1 kg and a scale up to 100 kg/f. We used the protocol proposed by Richards et al. (24), where participants were tested one by one in a seated position (standard position in a straight-backed chair). Each subject performed two attempts with each hand and one of the evaluators adjusted the dynameter to the grip size of the equipment. The hands performing the grips were alternated to minimize fatigue effects (1 to 2 min rest between each attempt). The best measurement was recorded for each of the two attempts. The TEM% between the two evaluations ranged from 1.2 to 1.5%.

### Statistics

The normality of the distribution of the data (weight, height and HGS) was verified by means of the Kolmogorov-Smirnov test. Descriptive statistics (mean and standard deviation) were calculated by age and sex strata. Significant differences between both sexes were verified by means of the Student's *t*-test for two samples. Different linear and nonlinear (cubic) regression analysis models were used, being the third degree cubic polynomial model the most adequate for both sexes (between age and HGS): HGS = a + b1(age) + b2(age)2 + b3(age)3, where: a is the intercept, b1, b2 and b3 are regression parameters, estimated from the data. The regression analysis was performed separately for each sex. For significance, *p* < 0.05 was adopted and calculations were performed in Microsoft Excel spreadsheets, SPSS 16.0 software and R. Percentile curves were created for HGS for both hands (P5, P15, P50, P85, and P9) using the LMS method [L (Lambda; skewness), M (Mu; median) and S (Sigma; coefficient of variation)] proposed by Cole et al. (25). The LMS Chart Maker software version 2.3 (26) was also used.

## Results

Table 1 shows the anthropometric characteristics, and HGS values of both sexes by age ranges. The boys categorized from 6 to 11 years of age presented similar values of weight, height, and HGS (right and left arm) in relation to their female counterparts (*p* > 0.05). On the contrary, in the age ranges (12 to 19 years, 20 to 29 years, 30 to 39 years, 40 to 49 years, 50 to 59 years, 60 to 69 and 70 to 80 years), males presented significant higher values in relation to females (*p* < 0.05).

**Table 1:** Characteristics of the sample studied.

**Age (years)**	***n***	**Weight (kg)**		**Height (cm)**		**HGS- RA(kgf)**		**HGS-LA (kgf)**	
		**X**	**SD**	**X**	**SD**	**X**	**SD**	**X**	**SD**
**Males**									
6 a 11	735	35.3	11.2	133.7	11.6	10.9	7.3	10.6	7.2
12 a 19	1534	64.8	13.9^*^	167.6^*^	9.8	31.7	13.7^*^	30.4	13.1^*^
20 a 29	322	77.3	12.3^*^	173.1^*^	6.7	43.8	12.3^*^	41.9	12.1^*^
30 a 39	49	82.9	12.8^*^	175.3^*^	9.4	33.4	17.4^*^	31.1	16.1^*^
40 a 49	43	84.3	15.4^*^	170.0^*^	5.5	27.2	12.7^*^	26.8	13.4^*^
50 a 59	53	86.9	11.6^*^	169.6^*^	6.8	25.8	11.1^*^	26.2	10.9^*^
60 a 69	52	81.5	13.0^*^	167.9^*^	6.6	29.4	15.3^*^	27.7	13.5^*^
70 a 80	52	78.6	11.7	165.6^*^	6	27.4	12.7^*^	25.3	13.0^*^
Total	2840	59.7	20.3	159.4	18.4	27.5	16.3	26.3	15.6
**Females**									
6 a 11	630	36.1	10.9	135.2	12.1	10.8	5.9	10.1	5.6
12 a 19	787	59.7	12.1	158,0	6.3	22.9	7.6	21.6	7.3
20 a 29	240	64.3	12.5	160.6	6.1	23.5	8.7	22,0	8.3
30 a 39	72	70.5	13.8	160,0	8.1	23.7	10.2	22.8	9.5
40 a 49	110	70.7	14,0	156.8	10.4	23.5	8.8	23,0	8.7
50 a 59	162	70.4	12.4	156.2	5.7	20.2	8.2	19.7	8.5
60 a 69	294	70.2	12.2	153,0	6.7	20.3	6.9	19.4	6.9
70 a 80	241	67.5	12.5	151.3	6.4	18.7	7.1	17.8	7.1
Total	2536	57.7	17.8	151.3	13.1	19.3	8.8	18.4	8.5

X, Average; SD, Standard deviation; HGS, Handgrip strength; RA, Right arm; LA, Left arm.

**Figure 1:**
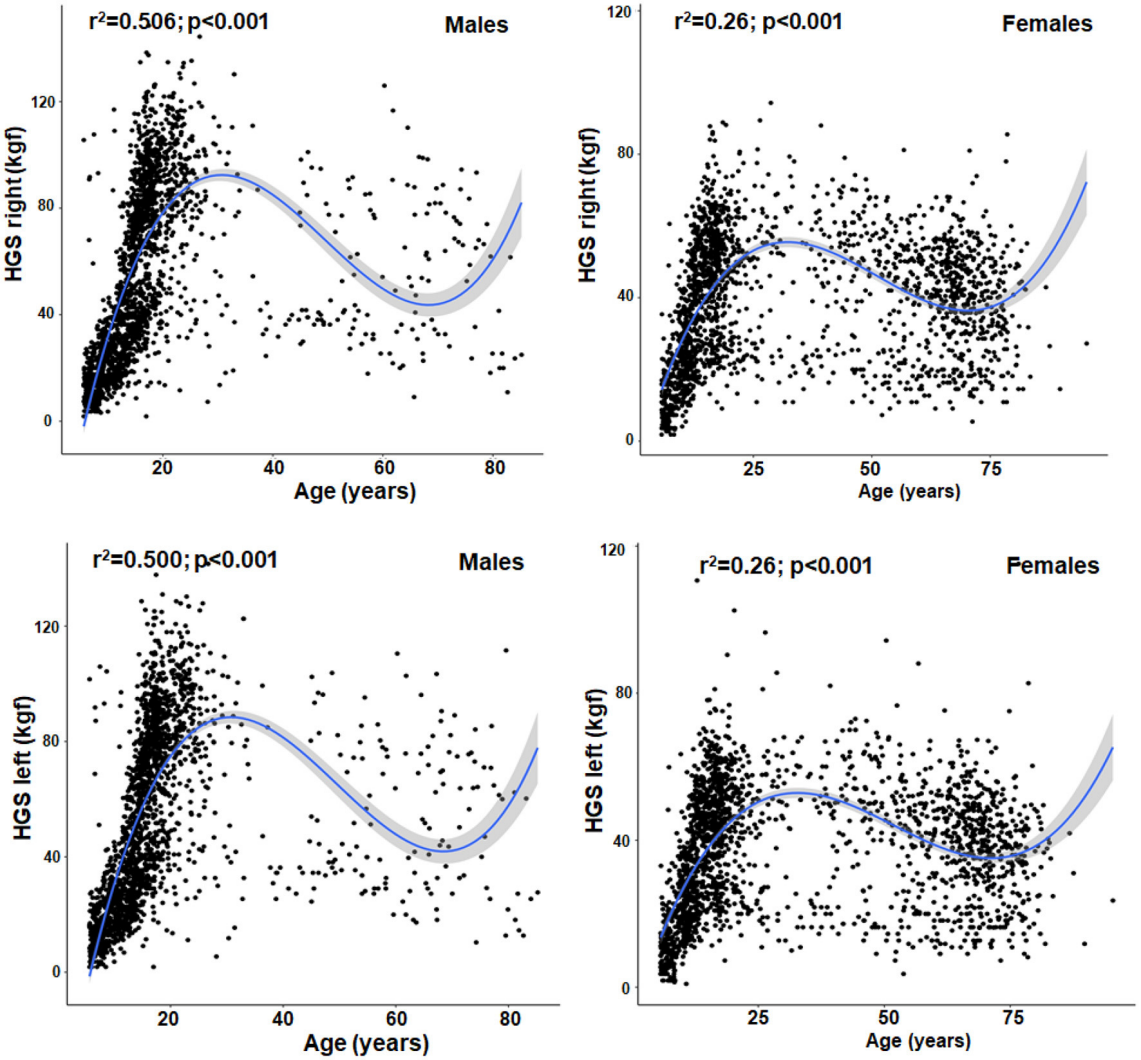
Non-linear relationship (cubic) between chronological age with HGS in both hands and both sexes from 6 to 80 years of age.

The results of the linear and nonlinear regression between age and HGS can be seen in Figure 1. In the linear relationship, age with HGS showed low explanatory power in both sexes (R^2^ = 0.07 in men and 0.02 in women). While when analyzed by the cubic (non-linear) model, the explanatory values increased ostensibly (in men up to R^2^ = 0.50 to 0.51 and in women up to 0.26). In the non-linear model generated, the residual standard error (RSE) reflected adequate fit, for example in males (RSE = 11.48 and 11.05), while in females (RSE = 7.55 and 7.34). In general, Figure 1 shows that the cubic (nonlinear) regression model showed a phase of accelerated increase in HGS in both sexes (during childhood, adolescence and young adulthood). Followed by a plateau around the age of 30 years approximately in both sexes (45 kg in men and 28 kg in women), then after the age of 40 years, men begin to experience an accelerated reduction of HGS up to ~50% at the age of 80 years. While in women a smaller reduction of HGS was observed as age advances (decreasing by ~37%, approximately).

**Figure 2:**
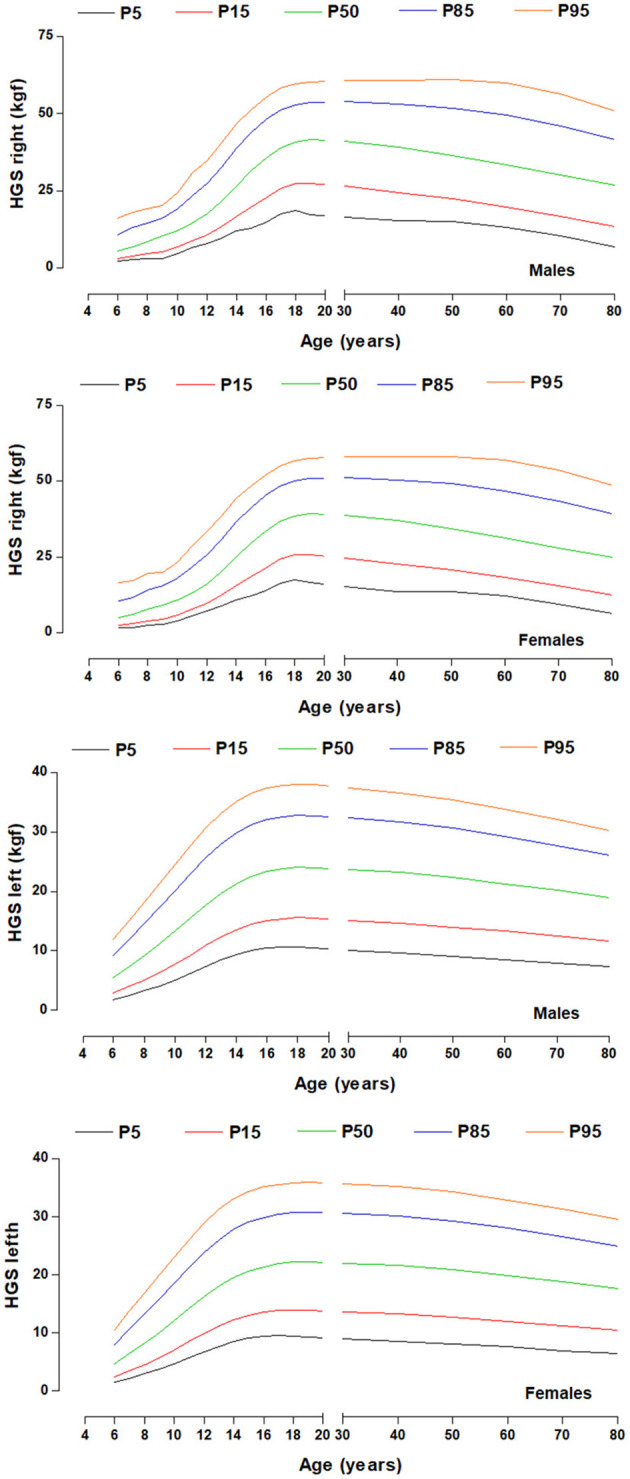
HGS percentiles by chronological age for both sexes and both hands.

The HGS percentiles by chronological age and sex can be seen in Table 2 and Figure 2. The percentile distribution was P5, P15, P50, P85, and P95. These values describe HGS patterns that are similar from age 6 to 12 years in both sexes and both hands. However, from the age of 13 years onwards, males present significantly higher levels of HGS (*p* < 0.05) than females until older ages.

**Table 2:** Percentiles to evaluate HGS from 5 to 80 years according to age range for both sexes.

**Age**	**HGS-RA (kgf)**								**HGS-LA (kgf)**							
	**L**	**M**	**S**	**P5**	**P15**	**P50**	**P85**	**P95**	**L**	**M**	**S**	**P5**	**P15**	**P50**	**P85**	**P95**
**Males**																
6	−0.09	5.56	0.62	2.1	3.0	5.6	10.8	16.3	0.41	5.52	0.55	1.8	2.9	5.5	9.2	11.9
7	−0.22	6.89	0.58	2.9	3.9	6.9	13.2	20.2	0.46	7.35	0.52	2.5	4.0	7.3	11.9	15.1
8	0.22	8.49	0.55	3.1	4.6	8.5	14.5	19.3	0.5	9.24	0.49	3.3	5.1	9.2	14.6	18.3
9	0.61	10.26	0.51	3.1	5.4	10.3	16.3	20.3	0.54	11.25	0.47	4.1	6.4	11.2	17.3	21.4
10	0.36	12.13	0.49	4.7	7.0	12.1	19.3	24.5	0.59	13.35	0.44	5.1	7.8	13.4	20.1	24.5
11	0.04	14.43	0.47	6.6	8.9	14.4	23.3	30.7	0.63	15.55	0.42	6.2	9.3	15.5	22.9	27.7
12	0.22	17.6	0.45	7.9	10.8	17.6	27.3	34.7	0.67	17.73	0.40	7.4	10.9	17.7	25.6	30.7
13	0.37	21.75	0.42	9.7	13.5	21.8	32.7	40.4	0.72	19.7	0.39	8.5	12.3	19.7	28	33.2
14	0.51	26.59	0.4	12.0	16.7	26.6	38.7	46.8	0.77	21.33	0.37	9.4	13.5	21.3	29.9	35.2
15	0.89	31.42	0.37	13.0	19.6	31.4	43.7	51.2	0.81	22.55	0.36	10.1	14.5	22.5	31.2	36.6
16	1.10	35.62	0.34	14.8	22.7	35.6	48	55.2	0.86	23.36	0.35	10.5	15.1	23.4	32.1	37.4
17	1.15	38.79	0.32	17.6	25.7	38.8	51.2	58.3	0.9	23.84	0.35	10.7	15.4	23.8	32.6	37.8
18	1.28	40.75	0.3	18.7	27.4	40.7	52.9	59.7	0.93	24.07	0.35	10.7	15.6	24.1	32.8	38.0
19	1.46	41.51	0.3	17.4	27.4	41.5	53.6	60.2	0.97	24.04	0.35	10.5	15.5	24.0	32.7	37.9
20 a 29	1.38	41.01	0.32	16.4	26.5	41	53.8	60.8	0.99	23.75	0.35	10.1	15.1	23.7	32.4	37.4
30 a 39	1.16	39.19	0.35	15.3	24.5	39.2	53	60.9	1.01	23.19	0.35	9.6	14.7	23.2	31.7	36.6
40 a 49	0.79	36.46	0.39	15.0	22.5	36.5	51.7	61.1	1.03	22.37	0.36	9.1	14.0	22.4	30.6	35.4
50 a 59	0.6	33.3	0.43	13.3	19.8	33.3	49.4	60	1.04	21.33	0.36	8.5	13.3	21.3	29.2	33.8
60 a 69	0.63	30.01	0.47	10.4	16.7	30.0	45.9	56.4	1.04	20.18	0.36	7.9	12.5	20.2	27.7	32.1
>70	0.78	26.69	0.51	6.8	13.5	26.7	41.6	51	1.05	18.99	0.37	7.3	11.7	19	26.1	30.3
**Females**																
6	−0.12	5.02	0.69	1.7	2.5	5.0	10.6	17.1	0.33	4.7	0.56	1.5	2.5	4.7	8.0	10.5
7	0.25	6.26	0.65	1.8	3.0	6.3	11.7	16.1	0.37	6.46	0.54	2.2	3.5	6.5	10.7	13.8
8	0.11	7.71	0.6	2.7	4.0	7.7	14.1	19.7	0.41	8.25	0.51	3.0	4.6	8.3	13.3	16.9
9	0.48	9.28	0.56	2.8	4.7	9.3	15.5	19.9	0.45	10.14	0.48	3.8	5.8	10.1	15.9	20.0
10	0.38	11.0	0.52	3.9	6.0	11.0	18.0	23.2	0.49	12.13	0.46	4.7	7.1	12.1	18.6	23.0
	**L**	**M**	**S**	**P5**	**P15**	**P50**	**P85**	**P95**	**L**	**M**	**S**	**P5**	**P15**	**P50**	**P85**	**P95**
11	0.13	13.2	0.49	5.6	7.8	13.2	21.7	28.6	0.53	14.2	0.44	5.8	8.5	14.2	21.3	26.1
12	0.15	16.25	0.47	7.2	9.8	16.2	25.9	33.5	0.58	16.25	0.42	6.8	9.9	16.3	23.9	28.9
13	0.35	20.26	0.44	8.9	12.4	20.3	30.8	38.5	0.63	18.08	0.4	7.7	11.2	18.1	26.1	31.3
14	0.51	24.95	0.41	11.0	15.5	24.9	36.5	44.3	0.68	19.57	0.39	8.5	12.3	19.6	27.9	33.1
15	0.86	29.61	0.37	12.3	18.5	29.6	41.3	48.5	0.74	20.68	0.37	9.1	13.1	20.7	29.1	34.4
16	1.11	33.63	0.34	14.0	21.5	33.6	45.3	52.0	0.78	21.43	0.37	9.4	13.6	21.4	29.9	35.2
17	1.17	36.65	0.32	16.5	24.3	36.7	48.3	55.0	0.83	21.9	0.36	9.6	13.9	21.9	30.4	35.6
18	1.28	38.51	0.3	17.5	25.8	38.5	50.1	56.6	0.86	22.15	0.36	9.5	14.0	22.2	30.7	35.9
19	1.39	39.23	0.31	16.6	25.9	39.2	51.0	57.4	0.89	22.18	0.37	9.3	13.9	22.2	30.8	36.0
20 a 29	1.33	38.75	0.33	15.2	24.8	38.8	51.2	58.0	0.91	21.98	0.37	9.0	13.7	22	30.6	35.7
30 a 39	1.17	37.02	0.36	13.7	22.8	37.0	50.4	58.0	0.93	21.56	0.38	8.6	13.3	21.6	30.1	35.2
40 a 49	0.81	34.41	0.4	13.6	20.8	34.4	49.1	58.1	0.93	20.88	0.38	8.1	12.7	20.9	29.3	34.3
50 a 59	0.61	31.35	0.44	12.2	18.4	31.4	46.8	57.0	0.93	19.94	0.39	7.6	12.0	19.9	28.1	32.9
60 a 69	0.61	28.14	0.48	9.6	15.6	28.1	43.5	53.7	0.92	18.82	0.39	7.0	11.3	18.8	26.6	31.3
>70	0.72	24.89	0.52	6.5	12.5	24.9	39.3	48.6	0.91	17.63	0.4	6.5	10.5	17.6	25.0	29.5

P, percentile; HGS, Handgrip strength; RA, Right arm; LA, Left arm.

## Discussion

The first objective of this study was to determine whether a nonlinear relationship model (cubic) could improve the prediction of HGS compared to the linear model by chronological age in a regional population of Chile ranging from 6 to 80 years of age. The results have shown that the nonlinear regression model (cubic) better explains HGS when it is analyzed by chronological age from infancy to senescence.

The cubic relationships observed in this study have evidenced a better explanatory power between age with HGS in both hands and in both sexes (reaching in men up to 51% and in women up to 28%). While, in the linear relationship, the predictive power for both hands showed values of R^2^ = 0.07 in men and 0.02 in women.

In both cases (for both right and left hand) the curvilinear (i.e., cubic) regression models were superior to linear regression as observed in some recent studies (27, 28). These cubic relationships observed in this study are consistent with the literature, where HGS increases with chronological age during growth and development. HGS reaches a plateau around age thirty (12, 29) and declines after age forty (10, 30) and fifty (28, 29), as age advances.

In essence, the predictive power of HGS improves when analyzed from a nonlinear relationship, as demonstrated in this study. Although several studies have used linear relationships between age and anthropometric parameters with HGS in various populations around the world (31–34). Therefore, future studies should take into account that to analyze HGS across the lifespan should consider that the cubic model can be a valuable tool to analyze upper limb muscle fitness trajectories.

Consequently, given that HGS is an indicator of general strength at all ages and stages of life, this study set out as a second objective to develop referential percentiles according to age and sex ranges.

The HGS percentiles as a function of chronological age proposed in this study should be interpreted using specific reference ranges according to geographic region and ethnicity (16). These proposed tools for the Maule region of Chile can be used to assess and monitor muscle strength from infancy to senescence.

In fact, regardless of the country and/or geographic region, the proposed references serve to identify individuals with low performance, to identify those in need of intervention, to follow up high-performing subjects as part of intervention programs (35). As well as to promote occupational therapy (13), physical education classes (12), and to identify frailty phenotypes and healthy aging across the lifespan (6).

In general, the cut-off points adopted in this study for HGS considered as abnormally low is <p5. These values were suggested by some studies (11, 36) where they highlight an early detection of decreased isometric muscle strength. Percentiles p5 to p15 can be interpreted with a low level of HGS and between p15 to p85 as adequate and >p85 as elevated HGS. Other studies have also pragmatically suggested 2SD below the mean maximum sex value (10, 37).

In general, reference values, by chronological age are a valuable tool to be considered as routine measures, both in clinical and epidemiological contexts. In fact, several international studies have used HGS as a prognostic marker of mortality in children, adolescents (38), in middle-aged people (39), and as indicators of frailty and sarcopenia in older adults (40).

This study has some limitations that have to do with the cross-sectional design used in this study, given that the results obtained preclude inferring causal relationships. In addition, data on muscle pathologies, muscle strength values of the lower extremities, including fat-free mass and bone mineral density values, were not collected. This information would have allowed a broader analysis and discussion of the results. Therefore, future studies should take these aspects into account.

Notwithstanding the above, we emphasize that one of the main strengths of the study is the size of the sample and the probabilistic selection, since the results obtained can be generalized to other geographic regions of Chile. These results can also serve as a baseline for future secular trend studies, as well as for short-term comparisons with other national and international studies. It can also serve professionals and researchers in the area as guidelines for clinical and epidemiological use and decision making. Furthermore, its use and application can enrich the International Classification of Functioning, Disability and Health [ICF] (41) in disciplines and sectors such as education and transportation, health and community services, respectively. Calculations can be made through the following link: http://www.reidebihu.net/hgs_infancy_senesce.php.

## Conclusion

In conclusion, this study demonstrated that there is a nonlinear relationship between chronological age and HGS from infancy to senescence. This cubic relationship allows us to observe a phase of accelerated increase in HGS during childhood and adolescence, until reaching a plateau around 30 years of age in both sexes, and consequently, a decrease in HGS from 40 years of age onwards, being more accelerated in men than in women. Furthermore, the proposed percentiles can serve as a guide for assessing and monitoring upper extremity muscle strength levels, as well as for surveillance, and in the planning of intervention programs at all stages of life.

## Data availability statement

The original contributions presented in the study are included in the article/supplementary material, further inquiries can be directed to the corresponding author.

## Ethics statement

The studies involving human participants were reviewed and approved by the UA 238-2014. Written informed consent to participate in this study was provided by the participants' legal guardian/next of kin.

## Author contributions

CU-A, FA-V, LC, and RV collected the data. MC-B, RG-C, MA, and ER participated on the conception and design of the study. MC-B, RG-C, JM, JS, and CT analyzed the data. All authors participated in the interpretation of the results, drafted and revised the manuscript, and approved the final revision of the manuscript.
